# Exceptionally omnidirectional broadband light harvesting scheme for multi-junction concentrator solar cells achieved *via* ZnO nanoneedles

**DOI:** 10.1038/srep39134

**Published:** 2016-12-14

**Authors:** Li-Ko Yeh, Wei-Cheng Tian, Kun-Yu Lai, Jr-Hau He

**Affiliations:** 1 Computer, Electrical and Mathematical Sciences and Engineering (CEMSE) Division, King Abdullah University of Science & Technology (KAUST), Thuwal 23955-6900, Saudi Arabia; 2 Graduate Institute of Electronics Engineering, National Taiwan University, Taipei 10617, Taiwan, ROC; 3 Department of Optics and Photonics, National Central University, Chung-Li 32001, Taiwan, ROC

## Abstract

GaInP/GaAs/Ge triple-junction concentrator solar cells with significant efficiency enhancement were demonstrated with antireflective ZnO nanoneedles. The novel nanostructure was attained with a Zn(NO_3_)_2_-based solution containing vitamin C. Under one sun AM 1.5G solar spectrum, conversion efficiency of the triple-junction device was improved by 23.7% *via* broadband improvement in short-circuit currents of 3 sub-cells after the coverage by the nanoneedles with a graded refractive index profile. The efficiency enhancement further went up to 45.8% at 100 suns. The performance boost through the nanoneedles also became increasingly pronounced in the conditions of high incident angles and the cloudy weather, *e.g.* 220.0% of efficiency enhancement was observed at the incident angle of 60°. These results were attributed to the exceptional broadband omnidirectionality of the antireflective nanoneedles.

Multi-junction solar cells hold great promise in the photovoltaic industry because of the high power conversion efficiencies (η’s) that are not achievable with their single-junction counterparts^1^,^2^,^3^. In particular, the multi-junction solar cell equipped with a concentrator system has delivered the record high η^4^. Since concentrator solar cells utilize focusing lens or tracking mirrors to collect sunlight in large areas, the output power generated by a unit cell area can be increased, leading to the reduced cost of cell materials^5^. To improve photovoltaic performances of the multi-junction devices, suppressing the undesired surface reflection, particularly at increased incident angles, is of crucial importance. In a solar concentration system, it is extremely difficult to avoid the deviation angle relative to the aligned optical path even with the state-of-the-art tracking apparatus^6^,^7^. The deviated light beams can lead to undesired efficiency loss owing to the low absorption at high incident angles^8^,^9^. One way to address the issue is to increase the omnidirectionality of reflection suppression on device surface. The antireflective (AR) surface with enhanced omnidirectionality can trap the photons from various incident directions through the scattering effect, in which light diffuse reflection is induced by the mircostructures or nanostructures properly fabricated on the surface^10^,^11^,^12^,^13^,^14^,^15^,^16^,^17^. The so-called light trapping effect greatly prolongs the optical path along the air/device interface, increasing the chance of optical absorption by the active region^11^.

Moreover, the induced light trapping effect can also benefits solar cells *via* the improved irradiance uniformity, which is strongly desired by the concentrator. The concentrated solar irradiance can easily damage the device by overheating the junctions^18^. Since the diffuse reflection triggered on the mircostructured or nanostructured surface can trap and redirect the incoming photons, the very high solar irradiance should be more evenly distributed on the device surface, preventing the thermal damage in localized areas.

Recently, with the advances in growth/fabrication techniques, the feasibility of omnidirectional and broadband suppression of surface reflection has been reported with many types of AR nanostructures^19^,^20^,^21^, bringing promising potential for single- and multi-junction solar cells^22^,^23^. The enhanced omnidirectionality of these nanostructured AR coating can further slash the cost of concentrator solar cells by making them less dependent on the pricy tracking system^7^,^8^. In addition to the aforementioned light trapping effect, the excellent AR properties of nanostructures also come from her capability of suppressing the reflection at long wavelengths. Since the nanoscale features become less resolvable in the long-wavelength solar spectrum, the nanostructured surface behaves as a transition layer from air to the device, breaking the abrupt transition of refractive index and thus facilitating optical transmission through the interface^24^. In general, increasing the grading of refractive index at the interface should render increased light absorption, but shaping the nanostructure with a certain desired feature proved to challenging. There have been numerous studies demonstrating superior photovoltaic performances of the devices with nano-engineered AR coating^25^,^26^,^27^. For example, it has been shown that GaAs single-junction solar cells covered with syringe-like ZnO nanorod arrays exhibit a 30% enhancement in conversion efficiency^25^. However, the results on multi-junction solar cells are much less found, let alone the studies with specifically shaped nanostructures and the characterizations under concentrated solar intensities and angle-dependent measurement.

In this work, a novel ZnO AR nanostructure is applied to commercial GaInP/GaAs/Ge triple-junction solar cells. The needle-like nanostructure is synthesized using a cost effective hydrothermal method, in which the diameter of a single rod can be gradually shrunk from the bottom to the top. Covered by the unique tapered nanorods, η of the tandem solar cell is effectively improved by 23.7% under normal incidence due to broadband improvement in short-circuit currents of all of 3 sub-cells. As the incident angle increases to 60°, the η enhancement goes up to 220%. Moreover, under the illumination of 100 suns, η of the tandem solar cell coated with the ZnO nanoneedles is 45.8% higher than that with bare surface. The excellent omnidirectionality and heat sustainability demonstrated here bring promising potential for multi-junction concentrator solar cells.

## Results

**Figure 1:**
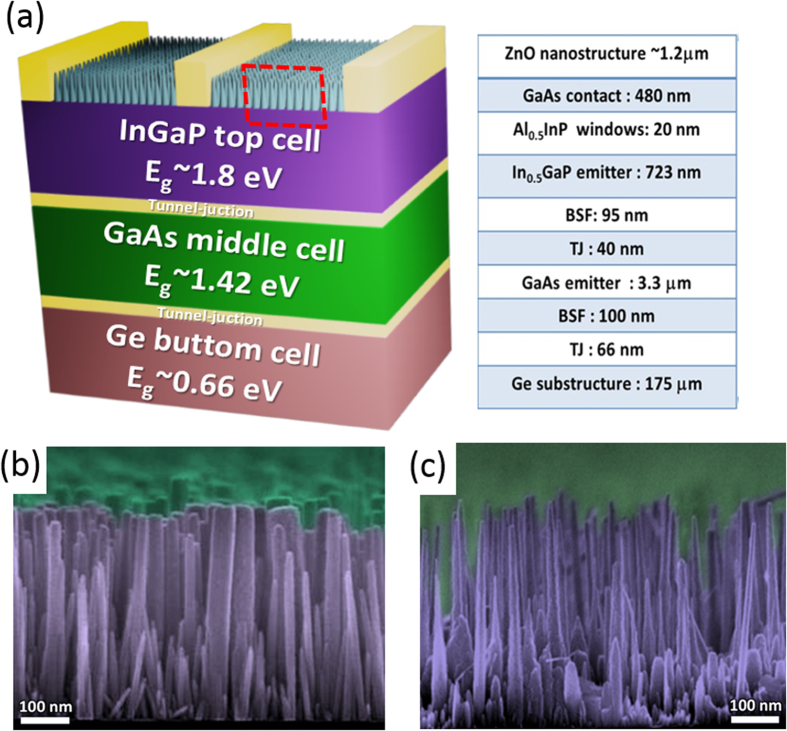
(**a**) Layer structure of the triple-junction solar cell. (BSF: back surface field; TJ: tunneling junction). The red dash-line rectangle encloses the ZnO AR nanostructure, which are displayed by the cross-sectional SEM images in (**b**) the nanorods grown without vitamin C; (**c**) the nanoneedles gown with vitamin C.

The ZnO nanostructures synthesized by the hydrothermal process are displayed by the scanning electron microscopy (SEM) images, as shown in the dotted line section of Fig. 1(a). Figure 1(b) and (c) present the nanorods grown without and with the addition of vitamin C, respectively. Comparing the two figures, it can be seen that adding vitamin C in the hydrothermal process leads to the tapered ends of the nanorods, exhibiting a needle-like feature. The tapered ends are attributed to the adhesion of vitamin C in the top region of the nanorods, which prevents the reaction of precursor in the proximity and thus results in gradual decrease in vertical/lateral growth rates of the nanorod^25^. The average length of the nanorods (Fig. 1(b)) and the nanoneedles (Fig. 1(c)) are both around 1.2 μm, and the base diameters seen in the two figures are all less than 100 nm. Since ZnO has a nearly-zero extinction coefficient and the refractive index of around 2.02 (an intermediate between those of air and the GaAs contact layer)^27^, the oxide nanostructure is expected to act as an effective AR medium.

**Figure 2:**
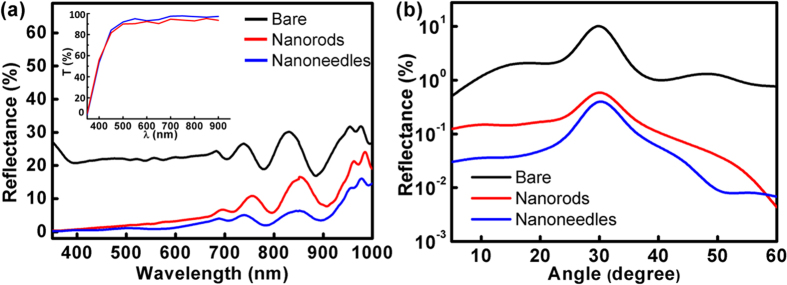
(**a**) Specular reflectance measured on the solar cells with different surface conditions. The inset is transmittance measured with the glass covered by ZnO nanorods and nanoneedls. (**b**) AOD-dependent reflectance on the devices with incident angle and wavelength fixed at 30° and 550 nm, respectively.

Figure 2(a) shows the spectra of specular reflectance (incident angle: 5°) measured on the solar cells with different surface conditions: the bare surface, the nanorods and the nanoneedles. The inset compares the transmittance of nanorod- and nanoneedle-covered glass slides. These measurements were performed with a standard UV-VIS spectrometer (JASCO ARN-733) equipped with an integrating sphere. One can see spectra oscillation occurring at the wavelengths above 700 nm, which is due to the fact that the device layer structure and the nanostructures are less resolved by the long wavelengths, and therefore interferences take place as light beams reflected at the layer interfaces. Evidently, the reflectance is considerably suppressed on the nanostructured surface. The suppressed reflectance mainly comes from the nanoscale dimension of the AR coating. Since the geometric features of the nanorods and the nanoneedles are mostly smaller than the studied wavelengths, the incident wave sees the nanostructure as an effective medium whose effective refractive index falls in between those of air and ZnO^25^. The effective medium provides a graded refractive index because of the increased space filling factor of ZnO toward the bottom, significantly reducing the undesired reflectance through destructive interferences among the beams reflected from different depths into the nanostructure^19^. As the nanoneedle arrays exhibit the most index grading from air to the device, the lowest reflectance (as well as the highest transmittance) is achieved.

The excellent AR property of the nanoneedles is also manifested at high-order scattering angles, which is demonstrated in Fig. 2(b). The figure presents the reflectance measured at the angle of detection (AOD, defined as the angle between surface normal and the reflected beam) ranging from 5° to 60°, with the incident angle fixed at 30°. The incident wavelength is selected to be 550 nm, being close to the peak of AM 1.5G solar spectrum power. It is obvious that the nanoneedles not only suppress the specular reflectance at 30°, but also render the greatly reduced reflectance at other scattering angles. One can further find that the extent of reflectance reduction becomes greater at increased AOD, indicating the exceptional light trapping capability of the nanoneedles^12^. The results shown in Fig. 2 suggest that the nanoneedles can capture much increased photons for the solar cell operating at high incident angles.

**Figure 3:**
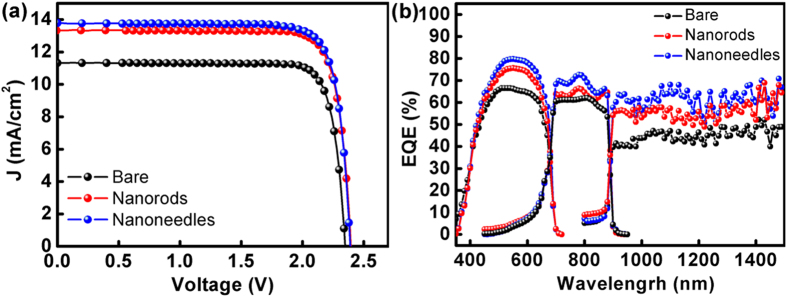
(**a**) J-V curves under AM 1.5G solar spectrum and (**b**) EQE spectra measured on the solar cells with the surface conditions of bare, nanorods, and nanoneedles.

Figure 3(a) shows the current density–voltage (J–V) curves under the solar illumination of one sun AM 1.5G (100 mW/cm^2^). Device characteristics derived from the curves are summarized in Table 1. It can be seen that the nanostructured AR coatings result in enhanced short-circuit current densities (J_sc_’s), indicating that the facilitated optical transmission into the device generates additional photocarriers. One can also see that covering the solar cells with ZnO nanorods or nanoneedles does not sacrifice the fill factor, which tells that surface passivation of the device is well maintained with the AR nanostructures. Overall, the enhanced J_sc_ brought by the nanoneedles without sacrificing other photovoltaic performances boosts the conversion efficiency of the solar cell by 23.7%, *i.e*. from 22.4% (of the bare surface) to 27.7%. Although the absolute efficiency of the triple-junction solar cell is not as high as other commercial products, which can be due to the unoptimized material qualities and fabrication conditions, the efficiency enhancement seen in Fig. 3(a) clearly shows that the ZnO nanostructure is effective in boosting the photovoltaic performances of multi-junction devices.

The effect of the nanostructures on photocurrent generation by each sub-cell can be analyzed with the external quantum efficiency (EQE) spectra, as presented in Fig. 3(b). The EQE spectra in wide wavelength range unambiguously demonstrate that the nanostructured AR coatings benefit carrier generation of all the three sub-cells. For example, the nanoneedles significantly raise the EQE at λ = 940 nm to 63.8% for the Ge bottom cell, which is 59% higher than that (40.2%) attained with the bare surface. Table 2 lists the short-circuit current densities of each sub-cell calculated by the following equation:


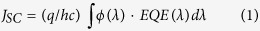


where ϕ(λ) is the intensity of AM 1.5G spectrum, EQE(λ) is the measured EQE obtained in Fig. 3(b), q is the electronic charge, λ is the wavelength of the monochromatic light, h is Planck constant, and c is the speed of light. It is found that the J_sc_ generated by the middle cell is lower than the J_sc_ listed in Table 1, which can be due to the variation of intensity distribution in the AM 1.5G spectrum used in the measurement. The results of Fig. 3(b) and Table 2 highlight an essential trait required by the AR coating for multi-junction solar cells: the wavelength range of reflection suppression must be broad enough to cover all the working ranges of each sub-cell. Since the J_sc_ under sunlight is limited by the cell delivering the least photocurrent, the AR structure merely promoting the EQE of a certain sub-cell does not promise the eventual η enhancement^28^. In other words, if only one or two of the three sub-cells shows promoted EQEs, the conversion efficiency under solar spectrum may still remain unchanged. As shown in Fig. 2(b), the increased index-grading of nanoneedles contributes to the most suppressed reflection over the studied wavelength range (350–1500 nm), in which EQE boosts are observed for all the three sub-cells, therefore a considerable η enhancement is achieved.

A close observation leads to the finding that EQE enhancement due to the nanostructure becomes less pronounced at the absorption wavelengths of the middle cell, such as λ = 730–750 nm and λ = 830–860 nm. The result should be discussed in consideration of the reflectance spectra shown in Fig. 2(a). Comparing the EQE and the reflectance spectra measured on the nanostructured surface, one can find that the wavelength ranges with less EQE enhancement correspond to those with interference oscillation peaks in the reflectance spectra, *i.e.* the EQE local minima occur at the wavelengths corresponding to reflectance local maxima. The reflectance peaks of the nanorods or nanoneedles compromise their AR performances, and thus result in the damped EQEs seen in Fig. 3(b). The results of Fig. 3(b) confirm that the superior broadband reflectance suppression by the nanoneedles is beneficial for the light harvesting of each sub-cell.

**Figure 4:**
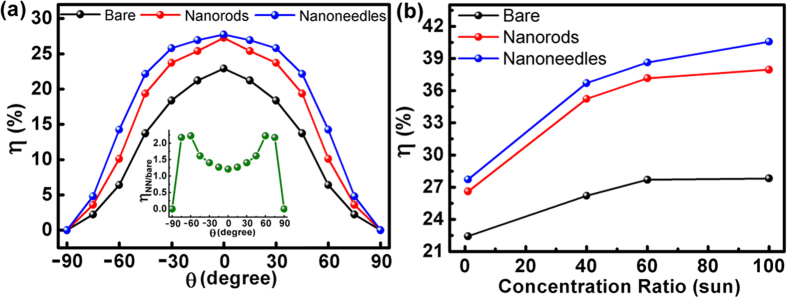
Conversion efficiencies of the triple-junction solar cells measured under AM 1.5G illumination as a function of (**a**) incident angle (under one sun); (**b**) solar concentrator ratio. The inset in (**a**) is η ratio of the nanoneedle device (η_NN_) to the bare one (η_bare_) plotted as a function of incident angle.

Figure 4(a) presents the η’s acquired with different incident angles. The measurement was carried out under one sun AM 1.5G solar simulator and the incident angle (θ) is that between the surface normal and the incident light beam, *i.e.* θ = 0° corresponds to normal incidence. One can see that η’s of the devices with nanorods or nanoneedles are apparently larger than that of the bare device at all θ’s. In particular, the η’s measured with the nanoneedles are fairly maintained at −30° < θ < 30°, whereas quick drop of η is seen with the other two devices as θ increases. The superior omnidirectionality of the nanoneedles becomes even clear in the inset, where the ratio (η_NN_/η_bare_) is plotted as a function of θ (η_NN_ and η_bare_ are η’s of the nanoneedle device and that of the bare one, respectively). At θ = ±60°, η_NN_/η_bare_ reaches the maximum of 2.2, indicating that a significant portion of the solar energy loss due to reflection can be recollected by the nanostructured surface. The much enhanced η at large θ’s roots in the fact that, compared to the cases of the other two surface conditions, the larger index-grading of the nanoneedles makes it easier for the incident light from all directions to reach the bottom of the nanostructure, as the light sees less dielectric chang. Since the dimensions of nanoneedles are increased in the bottom, the scattering (or light trapping) effect become increasingly pronounced, *i.e*. the incident photons are more likely to bounce back and forth among the nanoneedles. As a result, the optical path on the solar cell surface is prolonged, leading to enhanced absorption by the active region. Figure 4(b) shows the η’s of the three devices measured under the solar simulator with the concentration ratios up to 100 suns. It is found that all the η’s increase under concentrated illumination, which is due to the increased J_sc_ excited by the intensified solar energy and the logarithmic dependence of V_oc_ on J_sc_^29^:


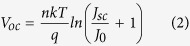


Where n, k, T, q and J_0_ are ideality factor, Boltzmann constant, temperature, electronic charge and the diode saturation current density, respectively. However, one can see that the η’s display a tendency of saturation at high concentration ratios, and the three solar cells exhibit saturated η’s at different solar intensities, *i.e.* η_bare_ almost stops climbing after 60 suns while η_NN_ still keeps rising at 100 suns. The less efficiency saturation of the nanoneedle-covered device further pushes the η enhancement to be 45.8% at 100 suns (in comparison with that of the bare device), demonstrating the excellent solar harvesting of the nanoneedles under concentrated illumination. The saturation of η comes from the inevitable junction heating brought by the high solar concentration, which degrades the photovoltaic device *via* the increased J_0_ and the consequent V_oc_ shrinkage^18^,^30^. Summarily, the evolution of η is dictated by the competition between J_sc_ and J_0_ as the concentration ration rises. Since the superior light trapping capability of the nanoneedles allows more incoming photons to be redirected on the surface and thus improve the irradiance uniformity over the active region, the undesired junction overheating under the nanoneedles is expected to be mitigated. With J_0_ being similar for the three solar cells in light of the same bulk material and device structure^13^, the largest J_sc_ contributed by the AR nanostructure defers the shrinkage of V_oc_ at elevated temperatures, and it is not surprising that the device with nanoneedles outperforms the other two at 100 suns.

**Figure 5:**
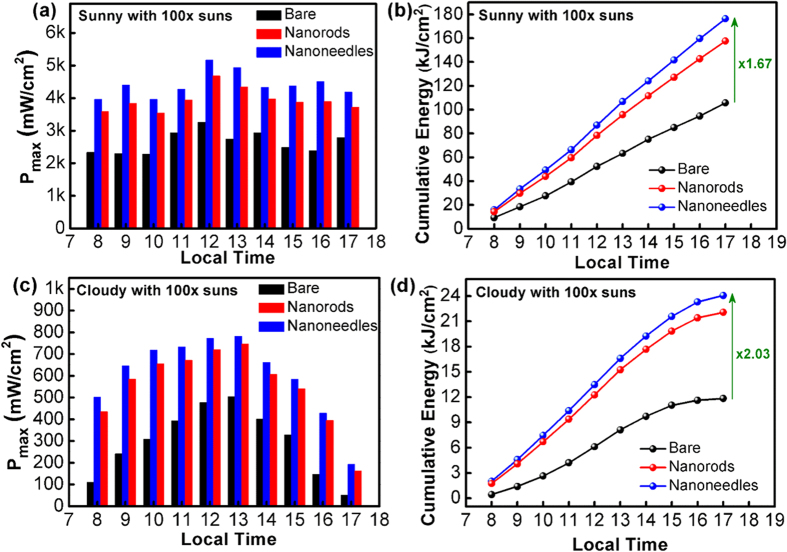
The maximum output power densities (P_max_) of the solar cells collected hourly with a 100X concentrator in: (**a**) the sunny day; (**c**) the cloudy day. The cumulative energy generated by the devices in: (**b**) the sunny day; (**d**) the cloudy day.

In order to evaluate the photovoltaic performances in real weather conditions, the triple-junction solar cells were characterized with a 100X concentrator in the sunny and the cloudy days. The characterizations were carried out at the latitude of 21.01 degree and the longitude of 121.53 degree. Figure 5(a) presents the maximum output power densities (P_max_) of the solar cells collected every hour from the 8^th^ hour to the 17^th^ hour (*i.e.* 5 pm local time Taipei, GMT+ 8) in the sunny day. It is clear that the device with nanoneedles delivers the largest P_max_ at all hours, being consistent with the measurement results under AM 1.5G solar simulator (shown in Figs 3(a) and 4(b)). Figure 5(b) displays the hourly cumulative energy calculated with the data in Fig. 5(a), where all the three devices exhibit linear increase in energy generation. At the 17^th^ hour, the solar cell with nanoneedle surface produces the energy of 176.4 kJ/cm^2^, which is 1.67-fold higher than that of the bare device.

Figure 5(c) and (d) show the results of the measurement repeated in the cloudy day. As expected, P_max_’s from the solar cells are considerably reduced with respect to those obtained in the sunny day. The result is ascribed to the decreased sunlight transmitted through the atmosphere, as well as the increased diffuse portion of solar irradiance. Since the diffuse portion of the sunlight in the cloudy condition is much increased, the photovoltaic performances attained in the cloudy day reflect the device capability of capturing the photons at high incident angles. In Fig. 5(d), it can be seen that the increase of cumulative energy starts to saturate after the 14^th^ hour, which is due to the continuous drop of P_max_ from that hour. More importantly, the superiority in light trapping brought by the nanoneedles is further magnified in the cloudy condition, as evidenced by the 2.03-fold enhancement of cumulative energy seen at the 17^th^ hour. This result clearly demonstrates the exceptional omnidirectionality of the AR nanoneedles.

## Conclusion

ZnO-based nanostructures were designed to improve light harvesting of triple-junction solar cells. It was found that the nanostructure with tapered tips, displaying a needle-like feature, renders the broadband light-harvesting performances superior to those exhibited by the flat-headed nanorods. The solar cell coated with the nanoneedles shows greatly improved photovoltaic performances, including the η enhancement from 22.4% to 27.7% and the EQE boost at the wavelengths ranging from 350–1500 nm. The characterizations under high incident angles, concentrated sunlight, and the cloudy weather conditions further confirm the superior omnidirectionality of the AR nanoneedles. These excellent AR performances are attributed to the sub-wavelength dimensions and favored geometry of the nanoneedles, which effectively suppress the surface reflection through the increased grading in refractive index from air to the device. The presented concept and manufacturing technique for AR structure should benefit high-efficiency photovoltaic devices in concentrator systems.

## Methods

### Preparation of the solar cells

The triple-junction solar cells were grown by metal organic chemical vapor deposition (Veeco TurboDisc E450 As/P system) with the layer structure shown in Fig. 1(a). The tandem solar cell comprises an InGaP top-cell, a GaAs mid-cell, and a Ge bottom cell, all of which are connected by Al0.1GaAs:Se tunneling junctions and monolithically grown on a p-type Ge substrate. The devices were fabricated with standard processes, including the top contact of Ti/Au realized by photo-lithography and e-beam evaporation, followed by the dry etching to define the mesa area of 4.5 × 5 mm^2^, and the deposition of Ge/Au to serve as the bottom contact.

### Synthesis of the ZnO nanoneedles

The ZnO nanostructure were synthesized using a one-step hydrothermal process. Before the hydrothermal growth, a 30-nm ZnO layer was deposited on device surface by room-temperature e-beam evaporation to serve as the seed layer for the nanorod growth. The device was then placed in the heated (95 °C) aqueous solution containing 10-mM zinc nitrate hexahydrate (Zn(NO_3_)_2_·6H_2_O, Sigma, 98% purity) and ammonia. The [0001] orientation of the nanoneedle is attributed to the intrinsic high energy of the O^2−^ terminated surface, to which the precursor molecules in the vicinity tend to be preferentially adsorbed. After 1.5 hours, the sample was removed from the solution, cleaned with ethanol and dried in air ambiance. The nanoneedles were attained by adding vitamin C in the solution. Since vitamin molecules tend to be adsorbed on the top ends of the nanostructures, nanoneedle growth is retarded by the hindered reaction between Z^2+^ and O^2−^ ions, which eventually leads to the shrunk diameters.

## Additional Information

**How to cite this article**: Yeh, L.-K. *et al*. Exceptionally omnidirectional broadband light harvesting scheme for multi-junction concentrator solar cells achieved *via* ZnO nanoneedles. *Sci. Rep.* **6**, 39134; doi: 10.1038/srep39134 (2016).

**Publisher's note:** Springer Nature remains neutral with regard to jurisdictional claims in published maps and institutional affiliations.

## Figures and Tables

**Table 1:** Device characteristics extracted from the J-V curves in Fig. 3(a).

AR Layers	V_oc_ (V)	J_sc_ (mA/cm^2^)	Fill Factor	η (%)
Bare	2.35	11.32	0.84	22.4
Nanorods	2.39	13.32	0.83	26.6
Nanoneedles	2.40	13.82	0.83	27.7

**Table 2:** The photocurrents of each sub-cell extracted from the EQE spectra in Fig. 3(b).

AR Layers	Top cell (mA/cm^2^)	Middle cell (mA/cm^2^)	Bottom cell (mA/cm^2^)
Bare	10.82	9.44	11.02
Nanorods	11.97	10.18	13.72
Nanoneedles	12.76	10.58	14.82
